# Evaluation of Potentially Inappropriate Medications Prescribed to Older Adults Upon Emergency Department Discharge

**DOI:** 10.1111/acem.70257

**Published:** 2026-03-09

**Authors:** Jessica Schowe, Andrew M. North, Kyle Schuchter, Katherine M. Hunold, Elizabeth Rozycki

**Affiliations:** ^1^ Department of Pharmacy The Ohio State University Wexner Medical Center Columbus Ohio USA; ^2^ Department of Pharmacy Eskenazi Health Indianapolis Indiana USA; ^3^ Department of Emergency Medicine, College of Medicine The Ohio State University Wexner Medical Center Columbus Ohio USA

## Abstract

**Background:**

Older adults are susceptible to adverse drug effects due to age‐related changes, a higher prevalence of comorbidities, and complexities in medication management. Nearly half of geriatric patients are prescribed at least one new medication at ED discharge. This study evaluated potential pharmacist interventions on ED discharge prescriptions for older adults using the Geriatric Emergency Medicine Safety Recommendations (GEMS‐Rx) list.

**Methods:**

This single‐center, IRB‐approved, retrospective review analyzed ED discharge prescriptions for potentially inappropriate medications based on GEMS‐Rx criteria from October 2021 to September 2024 for patients ≥ 65 years who were discharged from the ED. Prescriptions were reviewed by a trained pharmacist for medication‐related problems (MRPs). Outcomes included: rate of potential pharmacist intervention, number of prescriptions with at least one MRP, MRP types, missing risk vs. benefit documentation, rates of current practice pharmacist review, two or more GEMS‐Rx prescriptions at discharge, and polypharmacy. All prescriptions during the study period were reviewed to determine medication sub‐class distribution, with a random sample of 250 patients, ensuring at least 10 prescriptions per sub‐class, if available. Descriptive statistics were utilized.

**Results:**

During the study period, 1458 prescriptions were written for included sub‐classes. Of 284 prescriptions screened, 265 (for 250 patients) were included. The median (IQR) age was 69.5 (67–75) years with patients on a median (IQR) of 5 (3–8) scheduled home medications and discharged with a median (IQR) of 2 (1–3) new medications. Skeletal muscle relaxants (37.0%) and first‐generation antihistamines (28.7%) were most frequent. Pharmacist intervention was potentially needed in 204 patients (81.6.%) with a median (IQR) of 2 (1, 2) MRPs per patient. Common MRPs included dose adjustment (53.2%), indication mismatch (41.1%), and frequency (38.1%).

**Conclusions:**

Most GEMS‐Rx prescriptions had at least one MRP, indicating an opportunity for enhanced prescribing. Future research should target strategies to optimize medications at ED discharge for older adults.

## 1 Introduction

Older adults account for 20% of emergency department (ED) visits in the United States and their representation will continue to rise as the number of older adults is projected to increase from 58 million in 2022 to 82 million by 2050 [1]. Almost half of older adults are prescribed at least one new medication at ED discharge [2]. Prescribing new medications to older adults requires careful consideration due to pathophysiologic changes associated with aging that alter pharmacodynamic and pharmacokinetic properties, making medication response less predictable [3]. This population also has more chronic conditions and medications, thereby increasing their risk for adverse drug events and drug–drug interactions [4].

Due to these increased risks in older adults, several resources exist to aid clinicians. The 2023 American Geriatrics Society Beers Criteria provide guidance regarding potentially inappropriate medications (PIM) in older adults [5]. However, this focuses on chronic medications rather than commonly prescribed medications for acute ED conditions. For example, chronic use of opioids should be avoided in older adults with certain disease states or drug–drug interactions; however, a short course at discharge may be appropriate for select clinical indications. To address ED‐specific prescribing, Skains and colleagues published a consensus‐based list of potentially inappropriate medications for older ED patients, referred to as the Geriatric Emergency Medication Safety Recommendations (GEMS‐Rx) [6]. This list included eight medication classes to avoid or use with extreme caution when discharging older patients from the ED: benzodiazepines, skeletal muscle relaxants, barbiturates, first‐generation antipsychotics, first‐generation antihistamines, non‐benzodiazepine and benzodiazepine receptor agonist hypnotics, gastrointestinal stimulants, and sulfonylureas. Because the targeted populations for these two medication lists are different, there are some nuances between what may be considered potentially inappropriate; for example, the Beers criteria do not classify certain skeletal muscle relaxants (baclofen and tizanidine) as potentially inappropriate given that these are commonly used for spasticity conditions. The acute prescribing of these agents for musculoskeletal conditions from the ED warrants further considerations and thus was included on the GEMS‐Rx list.

While emergency medicine physicians play a key role in appropriate medication use for older adults, emergency medicine pharmacists also play a critical role reviewing and optimizing discharge prescriptions for older adults, particularly for high‐risk medications. Specifically, Cesarz and colleagues demonstrated a 10.1% intervention rate in adult and pediatric ED discharge prescriptions reviewed by an ED pharmacist, with 54% of interventions being made to prevent a medication error and 46% to optimize medication therapy [7]. Additionally, Lineberry and colleagues found an 18.5% intervention rate from ED pharmacists when a targeted discharge prescription review was implemented and integrated into the electronic medical record (EMR), with the most common interventions including change in dose or frequency [8]. While these studies targeted specific medication sub‐classes or all patients in the ED, the potential for pharmacy involvement on ED discharge prescriptions for high‐risk medications in older adults has not been studied. Therefore, this study's objective was to determine the potential for intervention on GEMS‐Rx medications prescribed to older adults ≥ 65 years old at ED discharge.

## 2 Methods

### 2.1 Study Design, Setting, and Population

This was a single‐center, retrospective chart review of discharge prescriptions in older adults following an ED visit from October 1st, 2021 to September 30th, 2024. This study was conducted at two EDs within the same health system in Columbus, Ohio, including an academic medical center with a Level I Geriatric Emergency Department Accreditation (GEDA) and a community hospital. Both departments are staffed by the same group of physicians and advanced practice providers. Together, these EDs account for over 125,000 patient visits annually, with geriatric patients accounting for approximately 24%.

Both sites had an existing process to alert EM pharmacists for real‐time discharge prescription review using the electronic medical record. Alerts were fired for high‐risk medication classes (anticoagulants, select anti‐infectives, anticonvulsants, anaphylaxis therapy agents, etc.), or patient‐specific factors (age greater than 80 years old or younger than 12 years old, serum creatinine greater than 1.5 mg/dL or less than 0.5 mg/dL, on hemodialysis, or weight less than 40 kg).

Patient prescriptions were included if they matched Medi‐Span subclasses aligned with the GEMS‐Rx list (Table 1) for a patient greater than or equal to 65 years of age who was discharged from the ED to a long‐term care facility or home. Prisoners, patients who were discharged from the ED by an inpatient service, and patients who had the discharge prescription canceled prior to discharge were excluded from review.

**TABLE 1:** GEMS‐Rx Class, Associated Medi‐Span Subclass, and Medication Names.

GEMS‐Rx class	Medi‐span subclass	Specific medication examples
Antihistamines (1st Generation)	Antianxiety Agents‐misc.	Hydroxyzine
	Antiemetics–Anticholinergics	Dimenhydrinate, Meclizine, Scopolamine
	Antihistamines–Alkylamines	Brompheniramine, Triprolidine, Dexbrompheniramine
	Antihistamines–Ethanolamines	Doxylamine, Diphenhydramine
	Antihistamines–Phenothiazines	Promethazine
	Antihistamines–Piperidines	Cyproheptadine
Antipsychotics (1st Generation)	Butyrophenones	Haloperidol
	Phenothiazines	Chlorpromazine, Fluphenazine, Perphenazine, Prochlorperazine
Barbiturates	Anticonvulsants‐misc.	Primidone, Brivaracetam^a^, Cannabidiol^a^, Carbamazepine^a^, Eslicarbazepine^a^, Gabapentin^a^, Levetiracetam^a^, Lacosamide^a^, Lamotrigine^a^, Pregabalin^a^, Oxcarbazepine^a^, Topiramate^a^, Zonisamide^a^
	Barbiturate Hypnotics	Phenobarbital
Benzodiazepines	Benzodiazepines	Alprazolam, Chlordiazepoxide, Clobazam, Clonazepam, Clorazepate, Diazepam, Lorazepam, Midazolam, Oxazepam
	Non‐Barbiturate Hypnotics	Estazolam, Temazepam, Triazolam
Gastrointestinal Stimulants	Gastrointestinal Stimulants	Metoclopramide
Non‐Benzodiazepine Receptor Agonist hypnotics	Non‐Barbiturate Hypnotics	Eszopiclone, Zaleplon, Zolpidem
Sulfonylureas	Sulfonylureas	Glimepiride, Glipizide, Glyburide
Skeletal Muscle Relaxants	Central Muscle Relaxants	Baclofen, Carisoprodol, Chlorzoxazone, Cyclobenzaprine, Metaxalone, Methocarbamol, Orphenadrine, Tizanidine

Abbreviation: GEMS‐Rx, Geriatric Emergency Medicine Safety Recommendations.

^a^ Non‐GEMS‐Rx medications included based on Medi‐Span subclass.

All discharge prescriptions during the study period were included in the data set pull, and a randomized sample of 250 patients was further reviewed ensuring there was representation for all medication subclasses. The distribution of medication subclasses in the entire sample determined the number of prescriptions reviewed per subclass, with a minimum of 10 prescriptions per subclass if available. This study was approved by the institution's Biomedical Institutional Review Board (IRB).

### 2.2 Outcome Measures

The primary outcomes were the rate of potential pharmacist intervention for discharge prescriptions based on the number of patients with at least one medication‐related problem (MRP). MRP types included indication, dose, frequency, duration, drug interaction, medication access barrier, monitoring or needed lab values, and education/counseling. MRPs were adapted from the Hanlon approach [9]. MRPs were assessed retrospectively in a standardized format by a trained reviewer evaluating the prescription for appropriateness and looking for all possible MRPs. The indication for prescribed medication was assessed based on ED clinical documentation and assessed for appropriateness based on GEMS‐Rx criteria and BEERS criteria if applicable as well as patient specific factors, such as comorbidities and previously used medications for indication. Dosing, frequency, duration, and drug–drug interactions were assessed for appropriateness using recommendations from Lexicomp for medication use in older adults. Drug–drug interaction was identified as an MRP if any category D or X drug–drug interaction was noted when the home medication list was assessed using Lexicomp. A sample of at least 20% was reviewed by a second senior reviewer for agreement. Discrepancies were discussed and adjudicated with the study team.

Secondary outcomes included the category and number of MRPs per prescription, lack of risk versus benefit documentation related to the medication selection, rate of pharmacist review through current institutional practice, rate of two or more GEMS‐Rx medications prescribed at discharge, and the rate of polypharmacy (defined as five or more medications at discharge) [10, 11]. Home medication lists were collected by counting the number of active, scheduled home prescription medications listed in the patient's prior to admission record for the encounter.

### 2.3 Statistical Analysis

Descriptive statistics were used to describe variables of interest, including counts and proportions for categorical variables and median (interquartile range) for continuous variables based on variable distribution. Data analysis was completed using STATA Statistical Software, version 16 (StataCorp LLP, College Station, Texas, USA).

## 3 Results

### 3.1 Characteristics of Patients and Prescriptions

**FIGURE 1:**
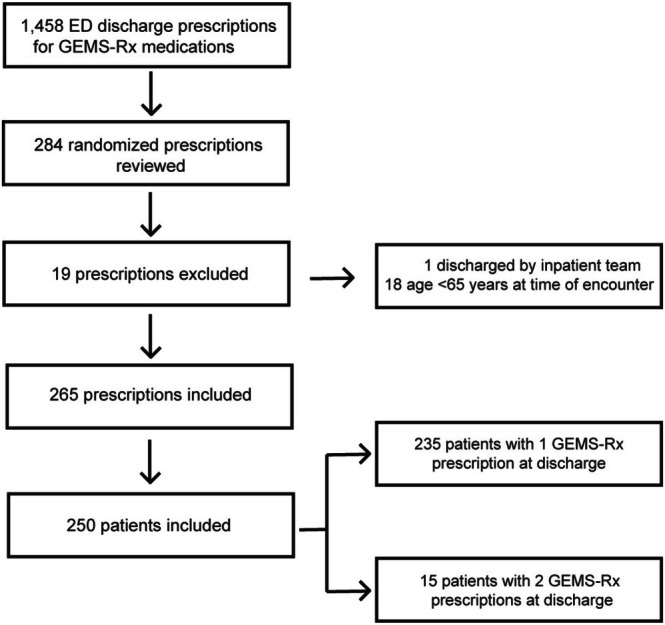
Patient and prescription sample selection.

During the study, 1458 prescriptions of included medication subclasses were written. Prescriptions were screened (*n* = 284) to include 250 patients (265 prescriptions) with potentially inappropriate medications (Figure 1). The median (IQR) age was 69.5 (67–75) years, and patients were on a median (IQR) of 5 (3–8) scheduled home medications prior to ED presentation. Patients were discharged with a median (IQR) of 2 (1–3) new medications. See Table 2 for additional baseline characteristics.

**TABLE 2:** Baseline characteristics of persons discharged from the ED with a GEMS‐Rx prescription.

Characteristic	Results (*N* = 250)
Age (Years), median (IQR)	69.5 (67–75)
Same‐Day ED Discharge, *n* (%)	181 (72.4)
ED Observation Status, *n* (%)	47 (18.8)
ED Location, *n* (%)	
Academic Site	143 (57.2)
Community Site	107 (42.8)
ISAR Score Included, *n* (%)	69 (27.6)
ISAR Score, median (IQR)	1 (0–2)
Total Number of Medications at Discharge, median (IQR)	7 (5–10)
Number of Home Scheduled Prescriptions, median (IQR)	5 (3–8)
Number of New Prescriptions at Discharge, median (IQR)	2 (1–3)
Medication Recommended By Consult Service, *n* (%)	33 (13.2)
CrCl < 60 mL/min, *n* (%), *N* = 162	77 (47.5)
At least one ED visit within past 6 months	113 (45.2)

Abbreviations: CrCl, creatinine clearance; ED, emergency department; IQR, interquartile range; ISAR, identification of seniors at risk.

### 3.2 Main Results

Skeletal muscle relaxants (37.0%) and first‐generation antihistamines (28.7%) were the most common GEMS‐Rx classes within the sample (Table 3). The primary outcome of rate of potential pharmacist intervention occurred in 218 patients (81.6%) with a median (IQR) of 2 (1, 2) MRPs per patient. Secondary outcomes such as pharmacist review through the current targeted discharge review process occurred in 22 patients (8.8%) and polypharmacy at discharge occurred in 189 patients (75.6%). Of the 22 reviewed by a pharmacist in real‐time, 3 prescriptions had pharmacist intervention (one dose reduction, one change to alternative medication, one accessibility issue).

**TABLE 3:** Distribution of prescriptions by GEMS‐Rx class in random sample versus total prescriptions.

GEMS‐Rx class	Random sample (*N* = 265)	Total prescriptions (*N* = 1458)
Skeletal Muscle Relaxants	98 (37.0)	614 (42.1)
First‐Generation Antihistamines	76 (28.7)	407 (27.9)
Barbiturates and Miscellaneous Anticonvulsants	48 (18.1)	320 (22.0)
Benzodiazepines	15 (5.7)	50 (3.4)
Metoclopramide	11 (4.2)	30 (2.1)
First‐Generation Antipsychotics	10 (3.8)	28 (1.9)
Sulfonylureas	6 (2.3)	7 (0.5)
Non‐Benzodiazepine Hypnotics	1 (0.4)	2 (0.1)

*Note:* Data presented as *n* (%).

Abbreviation: GEMS‐Rx, Geriatric Emergency Medication Safety Recommendations.

**FIGURE 2:**
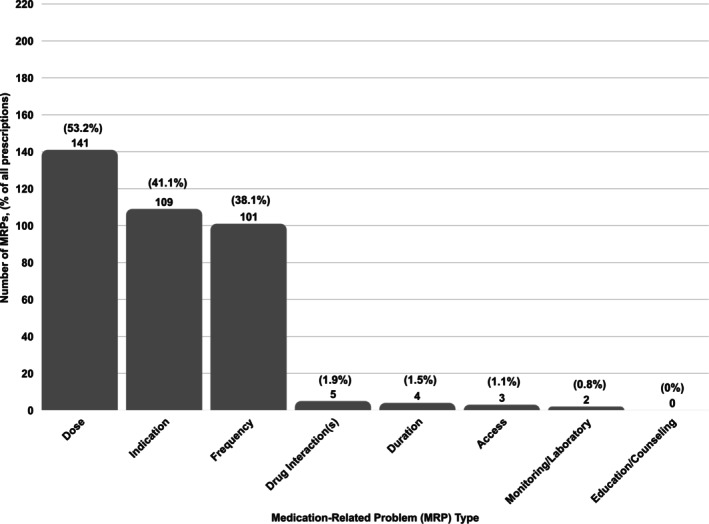
Rate of medication related problems by category.

Dose adjustment (53.2%), indication mismatch (41.1%), and frequency (38.1%) were the most common MRPs. The rate of MRPs by category is fully represented in Figure 2. Skeletal muscle relaxants, metoclopramide, and first‐generation antipsychotics had dose as the most frequent MRP. Frequency was the most common MRP for first‐generation antihistamines, barbiturates, and miscellaneous anticonvulsants. Benzodiazepines and sulfonylureas had indication as the most frequent MRP. A common finding was lack of risk versus benefit documentation to justify the use of a PIM in the medical record in 78.5% of patients. When considering differences between the two ED locations (academic and community sites), the rate of potential intervention was 78.3% at the academic site and 86.0% at the community site (*p* = 0.14).

## 4 Discussion

In this study, we found an opportunity for improved prescribing practices for high‐risk medications in older adults with potential ED pharmacist intervention on 81.6% of targeted medication prescriptions and at least 2 MRPs for most patients. While the study institution does hold Level I Geriatric Emergency Department Accreditation, indicating high‐quality geriatric care, our findings highlight the need for continuous quality assessment and protocol improvement as new data is available to continue to improve care for older adult ED patients. Further, the results demonstrate the importance of a multidisciplinary team approach in older adult patients.

Older adults face a heightened risk of adverse drug events due to age‐related changes in drug response and metabolism, which are further compounded by complex comorbidities that contribute to polypharmacy. Despite existing resources and focus on appropriate geriatric care in the study site ED, there are still opportunities for improvement when GEMS‐Rx medications are prescribed given the high rate of MRPs this study identified. Multiple opportunities exist to optimize prescribing practices at ED discharge to not only mitigate the MRP associated with each prescription but also to identify alternative therapies that may be preferred at discharge instead of a GEMS‐Rx prescription. To assess indication appropriateness in this study, reviewers used the GEMS‐Rx list of alternates to evaluate appropriate indications by examining alternatives trialed previously. This is just one example of a resource that may help guide appropriate pharmacologic treatment for older adults at ED discharge.

Furthermore, most patients had polypharmacy before their ED visit, defined both in this study and in GEDA guidance as five or more medications. Polypharmacy is associated with increased morbidity and mortality in older adults, possibly related to increased adverse drug events as well as increased hospitalizations [12]. Optimizing discharge prescriptions and avoiding MRPs can ease the burden that polypharmacy has on older adults, especially when alternatives to GEMS‐Rx medications are available. Emergency medicine physicians, advanced practice providers, and pharmacists are faced with the challenge to not only avoid MRPs but to prescribe optimized medications that are safe in this patient population.

The results of this study highlight the multifaceted approach necessary to implement systems to foster enhanced prescribing practices in older adults at ED discharge, with strategies varying by MRP type. A study by Elder and colleagues used clinical decision support to boost adherence to geriatric recommendations for a targeted list of 12 PIMs [13]. The authors found higher adherence to discharge prescription recommendations in the post‐implementation group (0.5% vs. 31.7%; difference 31.1%, 95% CI 27.5%–34.7%), further highlighting that clinical decision support tools are a valuable method to optimize drug therapy for older adults at ED discharge. While Elder and colleagues used the 2019 AGS Beers Criteria to identify a list of targeted medications and this current study utilized the GEMS‐Rx list, 10 of the 12 targeted medications for discharge prescription review overlapped. Clinical decision support can proactively guide optimal prescribing and can be considered when mitigating MRPs. For example, it may be more appropriate to have lower default doses and longer administration intervals for older adults. The configuration of clinical decision support can influence the selection of preferred medications based on indications within an older adult population.

Alongside clinical decision support, pharmacist review of ED discharge prescriptions is another tool to optimize prescribing practices. A systematic review and meta‐analysis by Skains and colleagues included nine studies highlighting pharmacist‐led medication review within the ED to identify PIMs [14]. These authors found that physicians and advanced practice providers adopted medication recommendations from clinical pharmacist review and that patients were also able to partially or fully uptake recommendations, such as discussing further with the prescriber. While the current study's institution does have a targeted EM pharmacist discharge prescription review process for all patients 80 years or older, less than 10% of the current study's medications were reviewed by a pharmacist at discharge. The selection of the 80‐year‐old age limit for the existing review process was based on workload feasibility. Utilizing a more targeted dual‐criteria of age plus a high‐risk medication class for older adults should be considered, as the study by Skains and colleagues demonstrates the role of EM pharmacists in medication selection for older adults at ED discharge.

Future implementation strategies should consider prescription volumes to evaluate feasibility for optimizing prescribing of medications for older adults at ED discharge. For example, during the study period there were over 30,000 discharge prescriptions from the study sites for older adults, with GEMS‐Rx medications accounting for less than 5% of these prescriptions (3.5% for academic Level 1 GEDA site and 4.1% for community hospital site). Implementation of clinical decision support encouraging lower dosages and more appropriate frequencies for older adults at the point of order entry may be more proactive in optimizing prescribing while utilization of a pharmacist‐review process may be a more reactive and time‐intensive approach. Additionally, education and guidance documents for specific indications and best prescribing practices in older adults should be considered on an ongoing basis.

## 5 Limitations

There were several important limitations of this study to consider. First, this study was a retrospective review without a comparator group. The findings are hypothesis generating in determining the best approach to intervene on different prescriptions. Furthermore, the clinical review was based solely on documentation and prescription information available in the EMR, which limited the ability to assess MRPs such as indication and risk vs. benefit documentation. In addition, patients may not have reported all home medications, or the medication history may not have been updated to reflect current home medications. Additionally, the home medication list was likely underestimated, as it only accounted for home medications that were not over the counter (OTC) or written for a frequency of as needed. Medi‐Span subclasses were utilized as this is how the electronic health record would be able to categorize the GEMS‐Rx classes; however, this may have resulted in the inclusion of some medications within the anticonvulsants‐misc subclass in order to comprehensively capture the barbiturate medications. While these medications are not explicitly called out by the GEMS‐Rx classes, the authors felt this represented a practice sample of what medications would be included based on electronic health record feasibility in the real world. Finally, the data set provided was based on prescription level data only so patient characteristics, such as age or comorbidities, in the total sample were not available to compare to the randomized sample.

## 6 Conclusion

When utilizing the GEMS‐Rx criteria to target ED discharge prescriptions, most prescriptions had at least one MRP indicating that the GEMS‐Rx criteria may be a high‐yield opportunity for interventions to improve prescribing either proactively by EHR decision support or targeted pharmacist review. Future research should explore various strategies to optimize medications at ED discharge for older adults.

## Author Contributions

Jessica Schowe: Writing – review and editing, writing – original draft, project administration, methodology, investigation, formal analysis, data curation, conceptualization. Andrew North: Writing – review and editing, methodology, conceptualization. Kyle Schuchter: Writing – review and editing, methodology, data curation, conceptualization. Katherine Hunold: Writing – review and editing, methodology, conceptualization. Elizabeth Rozycki: Writing – review and editing, writing – original draft, validation, supervision, methodology, investigation, formal analysis, data curation, conceptualization.

## Funding

The authors have nothing to report.

## Conflicts of Interest

The authors declare no conflicts of interest.

## Data Availability

The data that support the findings of this study are available on request from the corresponding author. The data are not publicly available due to privacy or ethical restrictions.
